# Poverty and food insecurity of older adults living in social housing in Ontario: a cross-sectional study

**DOI:** 10.1186/s12889-020-09437-3

**Published:** 2020-08-31

**Authors:** Melissa Pirrie, Leila Harrison, Ricardo Angeles, Francine Marzanek, Andrea Ziesmann, Gina Agarwal

**Affiliations:** 1 Department of Family Medicine, McMaster University, David Braley Health Sciences Centre, 100 Main Street West, Hamilton, Canada; 2 Department of Health Research Methods, Evidence and Impact, McMaster University, 1280 Main Street West, Hamilton, Canada

**Keywords:** Social determinants of health, Poverty, Food insecurity, Social housing, Older adults

## Abstract

**Background:**

Poverty and food insecurity have been linked to poor health and morbidity, especially in older adults. Housing is recognized as a social determinant of health, and very little is known about subjective poverty and food insecurity in the marginalized population of older adults living in subsidized social housing. We sought to understand poverty and food insecurity, as well as the risk factors associated with both outcomes, in older adults living in social housing in Ontario.

**Methods:**

This was a cross-sectional study using data collected from the Community Paramedicine at Clinic (CP@clinic) program. A total of 806 adult participants residing in designated seniors’ or mixed family-seniors’ social housing buildings attended CP@clinic within 14 communities across Ontario, Canada.

**Results:**

The proportion of older adults reporting poverty and food insecurity were 14.9 and 5.1%, respectively. Statistically significant risk factors associated with poverty were being a smoker (AOR = 2.38, 95% CI: 1.23–4.62), self-reporting feeling extremely anxious and/or depressed (AOR = 3.39, 95% CI: 1.34–8.62), and being food insecure (AOR = 23.52, 95% CI: 8.75–63.22). Statistically significant risk factors associated with food insecurity were being underweight (AOR = 19.79, 95% CI: 1.91–204.80) and self-reporting experiencing poverty (AOR = 23.87, 95% CI: 8.78–64.90). In those who self-reported being food secure, the dietary habits reported were consistent with a poor diet.

**Conclusion:**

The poverty rate was lower than expected which could be related to the surrounding environment and perceptions around wealth. Food insecurity was approximately twice that of the general population of older adults in Canada, which could be related to inaccessibility and increased barriers to healthy foods. For those who reported being food secure, dietary habits were considered poor. While social housing may function as a financial benefit and reduce perceived poverty, future interventions are needed to improve the quality of diet consumed by this vulnerable population.

## Background

Seniors, aged 65 and older, are projected to be 25% of Ontario’s population by 2041, compared to 16.4% in 2016 [1]. Amid this rising prevalence, food prices and the cost of living also continue to increase across Canada, causing deteriorating conditions for those with low incomes [2, 3]. According to Statistics Canada, the proportion of seniors living beneath the Low Income Measure (LIM), a common measure of poverty, has risen dramatically from 4.7% in 2000 to 12.1% in 2017 [4], which may be due to these rising costs of living [2–5]. The LIM is calculated based on the median income of the community in which the individual lives and is adjusted for their household size, therefore it is not the same threshold value for everyone. For seniors living alone, the percentage living in poverty increased from 12.1 to 30.5% over this same period, and living alone is three times more prevalent among female seniors than male seniors [4]. In 2017, an estimated 129,000 female seniors in Ontario were living alone and in poverty [4]. As a consequence, the Ontario Non-Profit Housing Association reported that the proportion of seniors on the waitlist for social housing, where rent is subsidized to typically equal 30% of household income, has increased from 21% in 2006 to 33% in 2014 [5]. This represents a substantial number of Ontario seniors residing in social housing currently and in the near future. Therefore, it is important to understand the rate of poverty in seniors who currently reside in social housing and their ability to afford basic needs, such as food, to inform future programs and policies.

Research has repeatedly demonstrated a direct link between poverty and poor health, morbidity, and mortality [6], but it is unknown whether social housing has an impact on this relationship. Older adults with a lower income are less likely to be physically active, less likely to access preventative health services, and more likely to engage in unhealthy behaviours, which results in higher healthcare costs [5, 7]. In Ontario, 60% of high-cost users of hospital and home care services are seniors [8]. However, safe and affordable housing is a key social determinant of health (SDoH) [6] and living in social housing may change the relationship between low income, health behaviours and health outcomes for seniors, but there is very limited research available.

Another key element of the SDoH is access to food and nutrition. Access to nutritious and affordable food is essential to maintaining a healthy lifestyle and preventing or managing chronic disease. Research has shown that food insecurity not only harms an individual’s health and well-being, but it is a major contributor to health care system costs [9]. Adults residing in food-insecure households have higher rates of chronic conditions, such as heart disease, diabetes, and depression [9]. In 2018, the prevalence of food insecurity among Canadian seniors was 2.6% [10]. A report found that among unattached, low-income adults, the risk of being food insecure was reduced by 50% once the individual reaches the age of 65 and becomes eligible for Canadian pension plans [11]. In Canada, there are additional financial benefit programs (i.e. Old Age Security [OAS] and the Guaranteed Income Supplement [GIS]) to assist low-income seniors once they reach the age of 65. Seniors who can access these programs have much lower rates of food insecurity compared to households that must rely on other sources of income [12]. Therefore, older adults living in social housing who are 55 to 64 years may be at increased risk for food insecurity.

Households that experience food insecurity not only lack access to food, they also lack housing, adequate food markets, transportation, food and nutrition knowledge, time for food preparation, and adequate income [9]. The causes of food insecurity are complex, however poverty is repeatedly demonstrated as the strongest predictor; as income declines, food insecurity rises [9].

Despite existing literature on food insecurity and poverty rates in seniors in Canada, there continues to be a gap in the literature on this information for seniors living in social housing in Ontario. This marginalized population is difficult for researchers to access and survey (e.g. low education and literacy) [13]. For this reason, this paper seeks to describe observed poverty and food insecurity rates in this hard-to-reach population and explore the related risk factors. Since many Ontario housing providers define “senior” for the purposes of housing eligibility as 55 years of age and over [13], this is the population that will be examined in this paper and the term “older adults” will be used. We hypothesize that poverty, food insecurity, and their risk factors will have higher rates in older adults living in social housing compared to the general population of older adults.

### Objectives

We sought to understand poverty and food insecurity rates, as well as the risk factors associated with both outcomes, in older adults living in social housing in Ontario, in order to inform and educate policy decisions with the ultimate goal of reducing health inequalities for this vulnerable population.

Specifically, this study sought to answer four research questions:

1. What is the estimated rate of self-reported poverty and food insecurity in an older adult social housing population in Ontario?
2. What are the risk factors associated with poverty in an older adult social housing population in Ontario?
3. What are the risk factors associated with being food insecure in an older adult social housing population in Ontario?
4. What are the dietary habits of those who self-report being food secure in this population?

## Methods

### Study design and setting

This was a cross-sectional study using data from the Community Paramedicine at Clinic (CP@clinic) program. CP@clinic is a health promotion and health prevention drop-in program that assesses modifiable risk factors for health conditions, educates participants, and then links them to community resources and back to their family doctor. The program is held weekly by community paramedics and is designed to target low-income older adults living in social housing buildings (rent-geared-to-income subsidized units). For a detailed description of CP@clinic, see the published protocol for the randomized controlled trial [14]. This study was approved by the Hamilton Integrated Research Ethics Board.

### Participants

The participants were older adults (aged 55 and older) residing in designated seniors’ social housing buildings or mixed family-seniors’ social housing buildings within 14 communities across Ontario, Canada (Frontenac County, Grey County, Guelph, Halton Region, Hamilton, Hastings Region, Hearst, Iroquois Falls, Matheson, Norfolk Region, Peel Region, Sudbury, Timmins, and York Region). In Ontario, the minimum age to be eligible for seniors’ social housing varies by community but can be as low as 55 [15]. All participants met with community paramedics for health assessments between 2018 and 2019. A total of 806 participants attended the CP@clinic program during this time frame and responded to questions related to SDoH (i.e. food insecurity and poverty).

### Measures

All measures were quantitative and pragmatically collected by community paramedics through their delivery of the CP@clinic program. The CP@clinic program database guides the paramedics through multiple assessments, including sociodemographic variables, self-reported health history (e.g. high cholesterol), modifiable chronic disease risk factors, fall risk, diabetes risk, and quality of life measures.

#### Poverty

In order to assess risk of or current experience of poverty, participants were asked: “Do you ever have trouble making ends meet at the end of the month? (Yes/ No).” This question comes from an evidence-based tool that was designed to identify poverty in primary care in Ontario [16].

#### Food insecurity

To assess risk of or current experience of food insecurity, the Brief Hunger Screening Tool was used [17]. Participants were asked: “In the past month was there any day when you or anyone in your family went hungry because you did not have enough money for food? (Yes/ No).” This single-question tool was designed to screen for hunger in primary care [17].

#### Risk factors

In addition to poverty and income insecurity assessment, health-related indicators were collected by paramedics. Physical measures included weight, height, and waist circumference; body mass index (BMI) was calculated (kilograms/metre^2^). Also collected were self-reported history of high cholesterol, diabetes, hypertension, heart attack, atrial fibrillation, transient ischemic attack, or stroke. In addition, current smoking status, alcohol use, fruit and vegetable consumption, high fat or fast food consumption, salt intake, physical activity, fall risk, self-reported general health, having a family doctor, and quality of life were collected. Health-related quality of life (HRQoL) was measured using five domains from the EQ-5D-3L: mobility, self-care, usual activities, pain/discomfort, and anxiety/ depression [18]. The Canadian Diabetes Risk Questionnaire (CANRISK) was also administered [19]. Sociodemographic factors collected were age, gender, ethnicity, education, marital status, and living alone.

### Statistical analysis

Descriptive statistics were performed. Multivariate logistic regression models were used to analyze associations for each of the two outcome variables (food insecurity and poverty) and variables collected through the CP@clinic program: modifiable health behaviours identified through the literature review of poverty in older adults (fruit and vegetable intake, high fat or fast food intake, smoking status), all sociodemographic variables (sex, age, education, ethnicity, and lives alone), all quality of life measures (social isolation score and the five domains of HRQoL), and two measures representative of general health (objectively measured BMI and self-reported general health). Only participants with complete data for these variables were included in the regression analyses. To reduce the number of variables in the model, and to account for small sample sizes observed in some response options, some variables were collapsed into fewer response categories. All analyses were completed with IBM SPSS Statistics 17.0.

## Results

A total of 806 participants were included in this study; see Table 1 for participant demographic information. The majority of participants were between the ages of 65 and 84 (64.1%), were female (69.5%), had some high school education or less (44.3%), lived alone (77.5%), and were white (74.8%), and the most common marital status was widowed (38.5%).

**Table 1:** Sociodemographic factors of study participants

Variable	All participants ***N*** = 806 n (%)
Demographics	
Sex (*n* = 746)	
Male	186 (23.1)
Female	560 (69.5)
Education (*n* = 792)	
Some high school or less	357 (44.3)
High school diploma	185 (23.0)
Any post-secondary education	250 (31.0)
Ethnicity (*n* = 806)	
White (both parents)	603 (74.8)
Other (One or both parents of other ethnicity)	203 (25.2)
Lives alone (*n* = 800)	
No	175 (21.7)
Yes	625 (77.5)
Age (*n* = 794)	
55–64 years	124 (15.4)
65–84 years	517 (64.1)
85 years and older	153 (19.0)
Marital status (*n* = 766)	
Single, never married	95 (11.8)
Common-law	12 (1.5)
Married	155 (19.2)
Separated	40 (5.0)
Divorced	154 (19.1)
Widowed	310 (38.5)

For health behaviours, participants most frequently reported being a non-smoker (84.5%), overweight or obese (65.1%), self-reported general health as good (42.8%), eating fruits and vegetables everyday (64.8%), and eating no high fat or fast foods per week (46.5%). For HRQoL, the majority of participants reported they had poor mobility (51.0%), no problems with self-care (80.1%), no problems with performing usual activities (67.0%), moderate-to-extreme pain or discomfort (63.4%), anxious or depressed (49.9%), and were not socially isolated (54.2%). See Table 2 for all participant health behaviour and HRQoL characteristics.

**Table 2:** Health behaviours, health-related quality of life, poverty, and food insecurity of participants

Variable	All participants ***N*** = 806 n (%)
Health Behaviours/Risk Factors	
Fruits and vegetables (*n* = 800)	
Not everyday	278 (34.5)
Everyday	522 (64.8)
Physical activity (*n* = 799)	
Less than 30 min per day	322 (40.0)
30 min or more daily	477 (59.1)
Alcohol drinker (*n* = 798)	
No	755 (93.7)
Yes	43 (5.3)
Salt added to food (*n* = 802)	
Always, often	205 (25.3)
Sometimes, rarely, never	597 (74.1)
Eats high fat or fast food (*n* = 801)	
Never	375 (46.5)
1–2 times/week	348 (43.2)
3 or more times/week	78 (9.7)
Currently smokes (*n* = 798)	
No	681 (84.5)
Yes	117 (14.5)
Has diabetes (*n* = 792)	
Yes	241 (31.1)
No/Not sure	551 (68.4)
Body Mass Index Category (*n* = 742)	
Normal	195 (24.2)
Underweight	22 (2.7)
Overweight/obese	525 (65.1)
Waist circumference risk category (*n* = 806)	
Low risk (≤37 inches for males, ≤31.5 inches for females)	287 (35.6)
Elevated risk (> 37 inches for males), > 31.5 inches for females)	519 (64.4)
Self-reported general health (*n* = 800)	
Poor	62 (7.7)
Fair	189 (23.4)
Good	345 (42.8)
Very good/ excellent	204 (25.3)
Has a family doctor (*n* = 806)	
No	84 (10.4)
Yes	722 (89.6)
Cardiometabolic disease indicator (*n* = 805)	
None	116 (14.4)
Has at least one of: TIA, stroke, heart attack, atrial fibrillation, high blood pressure, high cholesterol	689 (85.5)
At-risk for falling (*n* = 806)	
No	390 (48.4)
Yes	416 (51.6)
Diabetes risk category (CANRISK)^a^ (*n* = 551)	
Low	18 (3.3)
Moderate	141 (25.6)
High	392 (71.1)
Social isolation score (*n* = 806)	
≤3 (Negative)	437 (54.2)
4–6 (Positive)	269 (33.4)
> 6 (Positive, may have depression)	100 (12.4)
Health-related Quality of Life	
Mobility (*n* = 799)	
No problems	388 (48.1)
Any problems	411 (51.0)
Self-care (*n* = 797)	
No problems	646 (80.1)
Any problems	151 (18.7)
Usual activities (*n* = 800)	
No problems	540 (67.0)
Any problems	260 (32.3)
Pain/discomfort (*n* = 800)	
No problems	289 (35.9)
Any problems	511 (63.4)
Anxiety/depression (*n* = 788)	
No anxiety/depression	402 (49.9)
Moderate anxiety/depression	322 (40.0)
Extreme anxiety/depression	64 (7.9)
Outcomes	
Poverty (*n* = 804)	
No	684 (84.9)
Yes	120 (14.9)
Food Insecure (*n* = 804)	
No	763 (94.7)
Yes	41 (5.1)

*TIA* Transient Ischemic Attack; missing data ranged from 0 to 8%; ^a^ excludes individuals with diabetes

With respect to the study outcome of food insecurity, in the full sample (*n* = 806), 94.7% (*n* = 763) reported being food secure, 5.1% (*n* = 41) reported being food insecure, and 0.2% (*n* = 2) did not provide a response. There were 635 participants who had complete data for the variables included in the food security regression model; among these participants, 95.1% (*n* = 604) reported being food secure and 4.9% (*n* = 31) reported being food insecure.

For the study outcome of poverty, in the full sample (*n* = 806), 84.9% (*n* = 684) reported that they did not experience poverty, 14.9% (*n* = 120) reported experiencing poverty, and 0.2% (*n* = 2) did not provide a response. There were 635 participants who had complete data for the variables included in the regression model; among these participants, 85.7% (*n* = 544) reported that they did not experience poverty and 14.3% (*n* = 91) reported experiencing poverty.

Among those who self-reported being food secure (*n* = 763), 93.6% were non-drinkers, 73.5% said they sometimes or never added salt to their food, 47.2% said they ate no high fat or fast foods per week, and 33.4% reported that they did not eat at least one serving of fruits and/or vegetables every day.

Table 3 illustrates the significant factors associated with food insecurity through multivariate logistic regressions. Firstly, respondents who were underweight had approximate 19 times the odds of being food insecure, compared to those who had a normal weight (Adjusted Odds Ratio [AOR] = 19.79, 95% CI: 1.91–204.80). In addition, those who self-reported experiencing poverty had approximately 23 times the odds of being food insecure, compared to those who did not experience poverty (AOR = 23.87, 95% CI: 8.78–64.90). Both age and sex were not significantly associated with experiencing food insecurity in this model.

**Table 3:** Multivariate logistic regression of having self-reported food insecurity for all participants

	Food Insecure (***n*** = 604)versusFood Secure (***n*** = 31)		
**Variable**	**AOR**	**95%CI**	***P*-value**
Demographics			
Sex			
Male	REF	–	–
Female	0.44	0.15–1.26	0.127
Age			
55–64 years	REF	–	–
65–84 years	1.89	0.43–8.38	0.402
85 years and older	1.45	0.25–8.44	0.683
Education			
Some high school or less	REF	–	–
High school diploma	0.70	0.24–2.08	0.521
Any post-secondary education	0.57	0.19–1.72	0.318
Ethnicity			
White	REF	–	–
Other	0.76	0.22–2.71	0.676
Lives alone			
No	REF	–	–
Yes	3.37	0.64–17.81	0.152
Health Behaviours/Risk Factors			
Fruits and vegetables			
Not everyday	REF	–	–
Everyday	0.77	0.30–2.01	0.598
Currently smokes			
No	REF	–	–
Yes	1.24	0.42–3.71	0.700
Body Mass Index category			
Normal	REF	–	–
Underweight	19.79	1.91–204.80	0.012
Overweight/ obese	1.57	0.51–4.81	0.429
Self-reported general health			
Poor/ fair	REF	–	–
Good/ very good/ excellent	0.79	0.31–2.06	0.633
Eats high fat or fast food			
0 times/week	REF	–	–
1–2 times/week	1.38	0.49–3.88	0.538
More than 2 times/week	1.28	0.33–5.01	0.722
Social isolation score			
≤3 (negative)	REF	–	–
4–6 (Positive)	1.52	0.48–4.89	0.479
> 6 (Positive, may have depression)	2.53	0.63–10.10	0.190
Poverty			
No	REF	–	–
Yes	23.87	8.78–64.90	< 0.001
Health-related Quality of Life			
Self-care			
No problems	REF	–	–
Any problems	1.22	0.37–3.93	0.744
Usual activities			
No problems	REF	–	–
Any problems	1.88	0.61–5.87	0.275
Anxiety/depression			
No problems	REF	–	–
Moderate problems	0.49	0.15–1.55	0.224
Extreme problems	0.29	0.06–1.42	0.126
Mobility			
No problems	REF	–	–
Any problems	0.96	0.30–3.04	0.941
Pain/discomfort			
No problems	REF	–	–
Any problems	2.33	0.77–7.08	0.135

*AOR* Adjusted Odds Ratio, *CI* Confidence Interval

The factors associated with poverty through multivariate logistic regression are presented in Table 4. Respondents who smoked had approximately two times the odds of experiencing poverty compared to non-smokers (AOR = 2.38, 95% CI: 1.23–4.62). For those who self-reported feeling extremely anxious or depressed, they had three times the odds of experiencing poverty compared to those who were not anxious or depressed (AOR = 3.39, 95% CI: 1.34–8.62). Lastly, among those who self-reported feeling insecure had approximately 23 times the odds of experiencing poverty, compared to food secure (AOR = 23.52, 95% CI: 8.75–63.22). Both age and sex were not significantly associated with experiencing poverty in this model.

**Table 4:** Multivariate logistic regression of having self-reported poverty in all participants

Variable	Poverty (***n*** = 544) versus No Poverty (***n*** = 91)		
	AOR	95% CI	***p*** value
Demographics			
Sex			
Male	REF	–	–
Female	1.19	0.60–2.34	0.619
Age			
55–64 years	REF	–	–
65–84 years	0.93	0.43–2.01	0.861
85 years and older	0.81	0.31–2.10	0.660
Education			
Some high school or less	REF	–	–
High school diploma	1.69	0.88–3.26	0.117
Any post-secondary education	1.45	0.77–2.75	0.250
Ethnicity			
White	REF	–	–
Other	0.92	0.45–1.89	0.820
Lives alone			
No	REF	–	–
Yes	1.57	0.72–3.38	0.254
Health Behaviours/Risk Factors			
Fruits and vegetables			
Not everyday	REF	–	–
Everyday	0.60	0.34–1.05	0.075
Currently smokes			
No	REF	–	–
Yes	2.38	1.23–4.62	0.010
Body Mass Index category			
Normal	REF	–	–
Underweight	0.82	0.16–4.28	0.809
Overweight/ obese	0.75	0.41–1.39	0.360
Self-reported general health			
Poor/ fair	REF	–	–
Good/ very good/ excellent			
Eats high fat or fast food	0.84	0.47–1.50	0.550
0 times/week	REF	–	–
1–2 times/week	1.30	0.72–2.33	0.389
Social isolation score			
More than 2 times/week	2.05	0.91–4.62	0.084
≤3 (negative)	REF	–	–
4–6 (Positive)	1.29	0.69–2.44	0.427
> 6 (Positive, may have depression)	1.90	0.83–4.38	0.131
Food Insecure			
No	REF	–	–
Yes	23.52	8.75–63.22	< 0.001
Health-related Quality of Life			
Self-care			
No problems	REF	–	–
Any problems	0.87	0.39–1.95	0.735
Usual activities			
No problems	REF	–	–
Any problems	0.58	0.28–1.20	0.139
Anxiety/depression			
No problems	REF	–	–
Moderate problems	1.38	0.73–2.59	0.319
Extreme problems	3.39	1.34–8.62	0.010
Mobility			
No problems	REF	--	--
Any problems	1.74	0.93–3.26	0.082
Pain/discomfort			
No problems	REF	–	–
Any problems	0.99	0.55–1.79	0.972

*AOR* Adjusted Odds Ratio, *CI* Confidence Interval

## Discussion

In this study, the self-reported poverty rate was 14.9% in our sample of older adults living in subsidized housing buildings. Secondly, the self-reported food insecurity rate was 5.1%. When all risk factors were modeled together, being a smoker, being extremely anxious or depressed, and being food insecure were significantly associated with poverty. In addition, having an underweight BMI and self-reporting poverty were risk factors significantly associated with food insecurity. In those who self-reported being food secure, the dietary habits reported were considered to be consistent with a poor diet; for example, 42.7% reported eating high fat or fast foods 1–2 times per week and 33.4% did not eat fruits or vegetables every day.

Interestingly, the rate of self-reported poverty was much lower than expected. As the study sample was older adults living in subsidized housing, for which low income is an eligibility requirement, one would expect the poverty rate to be close to 100% if using a defined income threshold (e.g. LIM). However, the current study assessed poverty by the individual’s self-reported ability to “make ends meet” at the end of the month, which is a subjective measure of relative income [20]. It is possible that because individuals in subsidized housing are paying rent-geared-to-income (approximately 30% of income), they have leftover funds to spend on other expenses and this may contribute to relative feelings of wealth. In addition, when comparing themselves to others, individuals may not feel like they are experiencing poverty, as they perceive that others have comparable living conditions. Another possible explanation is the lack of social desirability in self-reporting poverty leading participants to have under-reported this attribute.

Consistent with the literature, our study showed smoking tobacco was a risk factor for poverty [13, 21]. Smoking is known to perpetuate poverty and being poor is also associated with a higher prevalence of smoking [21]. This stems from various factors, such as using smoking to help cope with difficult living conditions and having lower health literacy levels [13, 21]. Secondly, self-reporting extreme anxiety and/or depression were also significantly associated with poverty. Our previous work has shown that anxiety and depression are prevalent in this population [22]. According to the Canadian Mental Health Association, people struggling with mental illness often live in chronic poverty due to struggles with stigma and discrimination [23]; this can cause inadequate education and employment, and ultimately lead to inadequate income and poverty [2].

Secondly, we found that approximately twice as many older adults living in subsidized housing buildings self-reported experiencing food insecurity (5.1%) compared to the 2.6% prevalence of food insecurity among seniors in the 2012 Canadian Community Health Survey [10]. Although food banks exist to help in emergency situations, they often have limits on the number of times an individual can access them per year (e.g. once per month) [24]. In addition, individuals living in subsidized housing are not always able to access these support systems due to stigma associated with using food banks, feeling that personal food needs and preferences may not be met, and barriers to access such as distance to the food bank, lack of transportation, hours of operation, or lack of education about the existence of the food bank [25]. This demonstrates the need for targeted interventions to address these gaps.

Consistent with the literature, our findings demonstrate that poverty is the greatest risk factor for food insecurity [9, 10, 12]. In Canada, income is strongly associated with food insecurity [12]. As household income declines, the risk of being food insecure greatly increases [12]. This is often because housing costs utilize any money that would otherwise be allocated for food [26]. Because housing costs are often fixed monthly, food purchases are budget items that can be reduced. Secondly, we found that underweight BMI was associated with food insecurity. Having inadequate access to food logically means a lower intake of calories and thus a lower BMI.

Contrary to our hypothesis, age, sex, and living alone were not significant risk factors for poverty or food insecurity. Although older adults aged 55–64 tend to have more food insecurity compared to individuals aged 65 and above, as they are not yet eligible for age-restricted financial assistance programs (e.g. OAS, GIS) [11, 12], in this subsidized housing population, the odds of poverty and food insecurity were not significantly different across these two age categories. Similarly, senior women and seniors living alone tend to have greater prevalence of poverty and food insecurity [4, 11] but these were not significant in the current study. Therefore, the results suggest that providing housing with rent-geared-to-income acts as a financial benefit that can buffer inequities between these subpopulations; however, there is still a significant portion who are having difficulties making ends meet, experiencing food insecurity, and living with anxiety or depression.

Lastly, in those who reported being food secure, poor diet quality was observed. Food secure participants reported eating minimal fruits and vegetables per week and ate diets high in salt (25.8%) and high fat or fast foods, indicating lower nutrient intakes and consumption of fewer healthy meals. This is concerning because it has been established that poor diet is linked to developing chronic diseases, such as diabetes [27]. It may also explain why the rates of diabetes or risk of diabetes are so high in this population [15]. Although the majority of individuals self-reported not adding salt to their food, the high quantity of fast food being consumed suggests they may still have a high sodium diet. This is alarming when we consider the high rates of hypertension in this population [15] and could be a contributing factor in the development of hypertension. Modifying this health-related behaviour will be challenging in this population, already identified to have low education and low health literacy [13]. This finding also suggests that when evaluating food-related programs in low-income older adult populations (e.g. delivery of fruit and vegetable boxes), diet quality should be measured in addition to food security to understand the true impact of these interventions.

We hypothesize that the poor diet observed by this population could be partly due to low health literacy, as well as physical inaccessibility to amenities near the housing. It may be possible that our sample had poor availability of grocery stores that offered healthy and affordable food. Due to Canada’s current agricultural policies, high calorie and less nutritious foods are often much cheaper to purchase compared to healthier foods like fresh fruits and vegetables [28]. In addition, “food swamps,” which are neighbourhoods that have access to foods that are high in fat and calories, have been found to exist in areas of low socioeconomic status [29]. Thus, it is not only important to have access to food, but to have access to affordable nutritious food. The nutrition implications of these findings are not fully understood, and thus future research is needed to investigate this relationship further and more in-depth.

### Limitations

Firstly, due to the cross-sectional nature of the study, no causal inferences can be made about the data. For example, it is difficult to determine whether having a BMI that is underweight causes food insecurity, or whether food insecurity causes an underweight BMI. Secondly, due to social desirability, there may be self-reporting bias in our study. As both outcome variables were self-reported, it is possible that participants under-reported their experiences with poverty and/or food insecurity due to social desirability. In order to combat this limitation data collection was conducted by individuals who were unknown to the participant and not affiliated with the housing provider. Lastly, self-selection bias is likely to have occurred due to the sampling method. Advertisements were placed around the apartment buildings and residents were able to choose whether they attended the CP@clinic program, through which the current study data was collected. Thus, it is likely that the individuals more inclined to participate in the program were more mobile and healthier and may not be representative of the entire population of older adults living in subsidized housing buildings.

## Conclusions

As the Canadian population continues to age, so does the number of older adults expected to be living in subsidized housing buildings [5]. We found the poverty rate in this population to be lower than expected, which could be due to subsidized housing functioning as a type of financial benefit program, as well as the poverty measure being relative (not an absolute income threshold) and the problem of social desirability bias. The rate of food insecurity was approximately twice that of the general population of older adults in Canada, and for those who reported being food secure, poor diet quality was observed. These findings suggest that providing housing subsidies may play a role in reducing relative poverty in this low-income population, but there is still a need for improving food security and diet quality. Further research is needed to understand the barriers to accessing healthier foods and the nutritional implications of these findings in order to inform future policies and interventions specific to older subsidized housing residents.

## Data Availability

The data that support the findings of this study are not publicly available due to them containing information that could compromise participant privacy. De-identified, limited data will be shared by the lead author upon request.

## Abbreviations

BMI: Body Mass Index
CANRISK: Canadian Diabetes Risk Questionnaire
CP@clinic: Community Paramedicine at Clinic
GIS: Guaranteed Income Supplement
HRQoL: Health-Related Quality of Life
LIM: Low Income Measure
OAS: Old Age Security
SDoH: Social Determinants of Health
